# Stroke Care in the United Kingdom During the COVID-19 Pandemic

**DOI:** 10.1161/STROKEAHA.120.032253

**Published:** 2021-04-26

**Authors:** Abdel Douiri, Walter Muruet, Ajay Bhalla, Martin James, Lizz Paley, Kaili Stanley, Anthony G. Rudd, Charles D.A. Wolfe, Benjamin D. Bray

**Affiliations:** 1 School of Population Health and Environmental Sciences (A.D., W.M., A.B., M.J., A.G.R., C.D.A.W., B.D.B.), King’s College London, United Kingdom.; 2 Sentinel Stroke National Audit Programme (L.P., K.S.), King’s College London, United Kingdom.; 3 Department of Ageing Health and Stroke, Guy’s and St Thomas’ National Health Service (NHS) Foundation Trust and King’s College London, United Kingdom (A.B.).; 4 Royal Devon and Exeter NHS Foundation Trust, United Kingdom (M.J.).

**Keywords:** incidence, neuroimaging, quality of health care, registries, stroke

## Abstract

The coronavirus disease 2019 (COVID-19) pandemic has potentially caused indirect harm to patients with other conditions via reduced access to health care services. We aimed to describe the impact of the initial wave of the pandemic on admissions, care quality, and outcomes in patients with acute stroke in the United Kingdom.

**Methods:**

Registry-based cohort study of patients with acute stroke admitted to hospital in England, Wales, and Northern Ireland between October 1, 2019, and April 30, 2020, and equivalent periods in the 3 prior years.

**Results:**

One hundred fourteen hospitals provided data for a study cohort of 184 017 patients. During the lockdown period (March 23 to April 30), there was a 12% reduction (6923 versus 7902) in the number of admissions compared with the same period in the 3 previous years. Admissions fell more for ischemic than hemorrhagic stroke, for older patients, and for patients with less severe strokes. Quality of care was preserved for all measures and in some domains improved during lockdown (direct access to stroke unit care, 1-hour brain imaging, and swallow screening). Although there was no change in the proportion of patients discharged with good outcome (modified Rankin Scale score, ≤2; 48% versus 48%), 7-day inpatient case fatality increased from 6.9% to 9.4% (*P*<0.001) and was 22.0% in patients with confirmed or suspected COVID-19 (adjusted rate ratio, 1.41 [1.11–1.80]).

**Conclusions:**

Assuming that the true incidence of acute stroke did not change markedly during the pandemic, hospital avoidance may have created a cohort of untreated stroke patients at risk of poorer outcomes or recurrent events. Unanticipated improvements in stroke care quality should be used as an opportunity for quality improvement and to learn about how to develop resilient health care systems.

## Abstract 2

Supplemental Digital Content is available in the text.

Concern that the coronavirus disease 2019 (COVID-19) pandemic might overwhelm health services in the United Kingdom led to rapid decisions to create additional hospital capacity for infected or suspected COVID-19 patients primarily by reducing elective hospital treatments, early discharge of patients who could be managed in other settings, and advising the public only to present to hospital in case of real need. Experience from countries that had already seen large numbers of COVID-19 cases suggested that such measures had an adverse impact on the delivery of emergency and specialist care for non–COVID-19 patients. In Shanghai, China it was reported that the stroke thrombectomy rate fell by about 50%,^1^ and using data from imaging software analysis systems in the United States, the number of urgent stroke-related brain scans fell by 39%.^2^ Data from the United Kingdom^3^ showed that in March 2020, at the beginning of the first wave of severe acute respiratory syndrome coronavirus 2 infections, attendances at emergency departments fell by 29%.

Between 80 000 and 100 000 people with stroke are admitted to hospitals in England, Wales, and Northern Ireland each year. The United Kingdom has well-established stroke services, and stroke has been a major focus for health policy over the past 10 years.^4,5^ One of the consequences has been to establish networked stroke services with centralization of specialist acute stroke care in specific hospitals to ensure that all patients are managed by adequately staffed and equipped stroke teams.^6^

The aim of this study is to describe the impact of the initial phase of the COVID-19 pandemic on acute stroke admissions, quality of care, and outcomes using data from a nationwide quality registry in England, Wales, and Northern Ireland.

## Methods

### Data Source

Data were collected by the Sentinel Stroke National Audit Programme (SSNAP)—the national quality register for stroke care that includes all hospitals admitting patients with acute stroke in England, Wales, and Northern Ireland (covering 92% of the population of the United Kingdom). Data are submitted prospectively on all patients presenting with acute stroke by clinical teams using a secure electronic case report form from the time of admission up to 6 months after stroke and include data on demographic and clinical characteristics, treatments, and outcomes. Overall case ascertainment of SSNAP pre–COVID-19 is estimated to be 95% of all acute stroke admissions.^7^ Data are available on request to SSNAP and the Healthcare Quality Improvement Partnership, and the study protocol is available on request to the corresponding author.

### Study Design

We performed a prospective registry-based cohort study of adult (age, ≥18 years) patients admitted to hospital with acute stroke (ischemic, primary intracerebral hemorrhage or undetermined type). Both patients with out-of-hospital and in-hospital strokes were included. The index date was the date of admission for patients with stroke onset outside of hospital or the date of stroke onset for patients having an acute stroke while already a hospital inpatient. Patients were eligible for inclusion if their index date was between October 1, 2019, and April 30, 2020, or equivalent time periods in 2016-2017, 2017-2018, or 2018-2019. Intensive population-level viral transmission control measures were introduced in the United Kingdom (lockdown) on March 23, 2020, mandating social distancing measures and the closure of most schools, workplaces, and retail and recreational facilities. Patients admitted during the initial lockdown period to April 30 were compared with patients admitted in the equivalent historical control periods (2017, 2018, and 2019). Patients were included in the study if they were admitted to a hospital continuing to prospectively submit data to SSNAP during the lockdown period.

### Statistical Analysis

Descriptive statistics were performed for patient demographics (age at the time of stroke, sex, and ethnicity), clinical characteristics (stroke type, onset in or out of hospital, time from onset to admission, prestroke modified Rankin Scale score, and National Institutes of Health Stroke Scale score), interventions and care quality metrics (stroke unit within 4 hours of arrival at hospital, brain imaging within 1 hour, intravenous thrombolysis and door to needle time, mechanical thrombectomy rate, swallow screening, time to stroke specialist nursing and physician review, and therapy assessments), and outcomes (all-cause inpatient mortality within 7 days of admission and modified Rankin Scale score at discharge from hospital). Comparisons between categorical variables were performed using χ^2^ tests. Changes over time in case fatality were analyzed as an interrupted time series using segmented log-linear regressions. These models used a joinpoint permutation test to select the optimal model that best fitted the data and tests of significance using a Monte Carlo method.^8^ The relative rate of mortality was modeled using Poisson regression using a robust variance estimator and was adjusted for age and National Institutes of Health Stroke Scale. All statistical analyses and graphical presentations were performed using R, version 3.6.3. A completed RECORD Checklist (Reporting of Studies Conducted Using Observational Routinely-Collected Data) is included in the Data Supplement.

### Ethical Approval

Permission for SSNAP to collect patient data without explicit consent was granted by the Confidentiality Advisory Group of the Health Research Authority under Section 251 approval.

## Results

A total of 203 653 patients with confirmed acute stroke were admitted during the October-April periods across the study’s 4 consecutive years period. Of these, 184 017 (90.3%) patients admitted to continuously participating hospitals were included in the analysis.

During the initial lockdown period, 114 of 130 hospital trusts (88%) continued to submit data for a total of 6923 stroke admissions, of which 5704 were followed up until death or discharge from hospital. The same 114 trusts admitted an average of 7902 (SD, ±423) stroke patients during the equivalent period in the previous 3 years. The 114 hospital trusts continuing to submit had a similar geographic distribution and volume of admissions to the 130 trusts submitting pre–COVID-19 (Table I in the Data Supplement).

**Figure 1.**
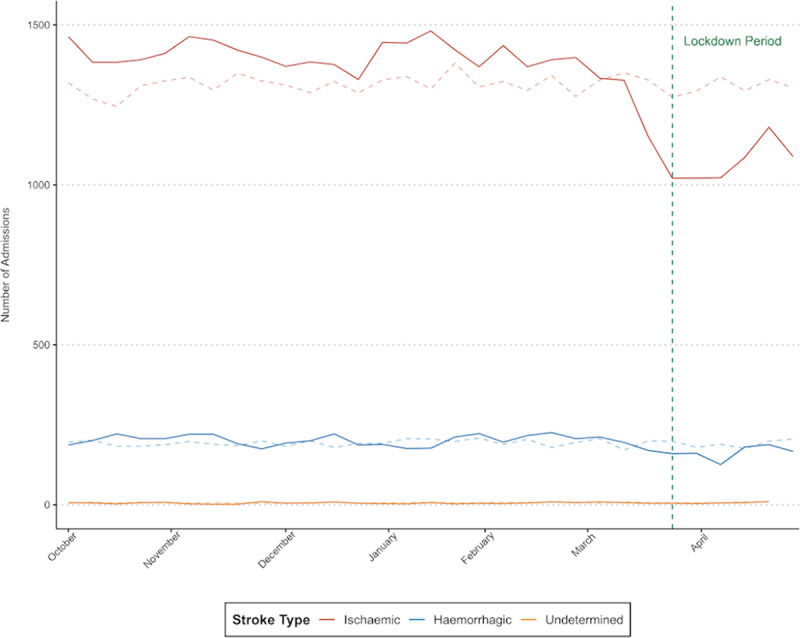
Weekly number of admissions for ischemic stroke, primary intracerebral hemorrhage, and undetermined stroke from October 1, 2019, to April 30, 2020, compared with the 3 previous years (dashed lines).

The number of admissions remained stable (estimated weekly percentage change of −0.05% [95% CI, −0.42 to 0.33]) up until the second week of February when there was a steep decline in the number of admissions (−3.10% [95% CI, −4.14 to −2.03]; *P*<0.001 for change in slope; Figure I in the Data Supplement). During the lockdown period, there was a 12.4% reduction in the overall number of stroke admissions compared with the mean during historical control periods (6923 versus 7902), but this reduction was only statistically significant for ischemic stroke and not for primary intracerebral hemorrhage or undetermined stroke (Figure 1). This reduction in admissions was associated with age and stroke severity, with proportionally larger falls in admissions for patients aged over 65 years and for patients with less severe strokes (National Institutes of Health Stroke Scale score, 0 and 1–4; Figures II and III in the Data Supplement).

Patients admitted during the lockdown period were broadly similar to historical controls (Table 1), but there were modest differences in some patient characteristics. There was a lower proportion of patients aged ≥85 years (19.7% versus 22.6%; *P*<0.001), fewer patients with White ethnicity (84.8% versus 87.9%) but more with unreported ethnicity (8.9% versus 5.8%; *P*<0.001), and a lower prevalence of atrial fibrillation (18.2% versus 19.6%; *P*=0.009) and prior stroke or transient ischemic attack (24.4% versus 25.7%; *P*=0.030). There was evidence of a shift in the distribution of prestroke functioning with a lower proportion of patients with higher modified Rankin Scale scores and a shift toward greater stroke severity in the lockdown period (16.7% versus 14.9% with National Institutes of Health Stroke Scale score >15; *P*<0.001).

**Table 1.**
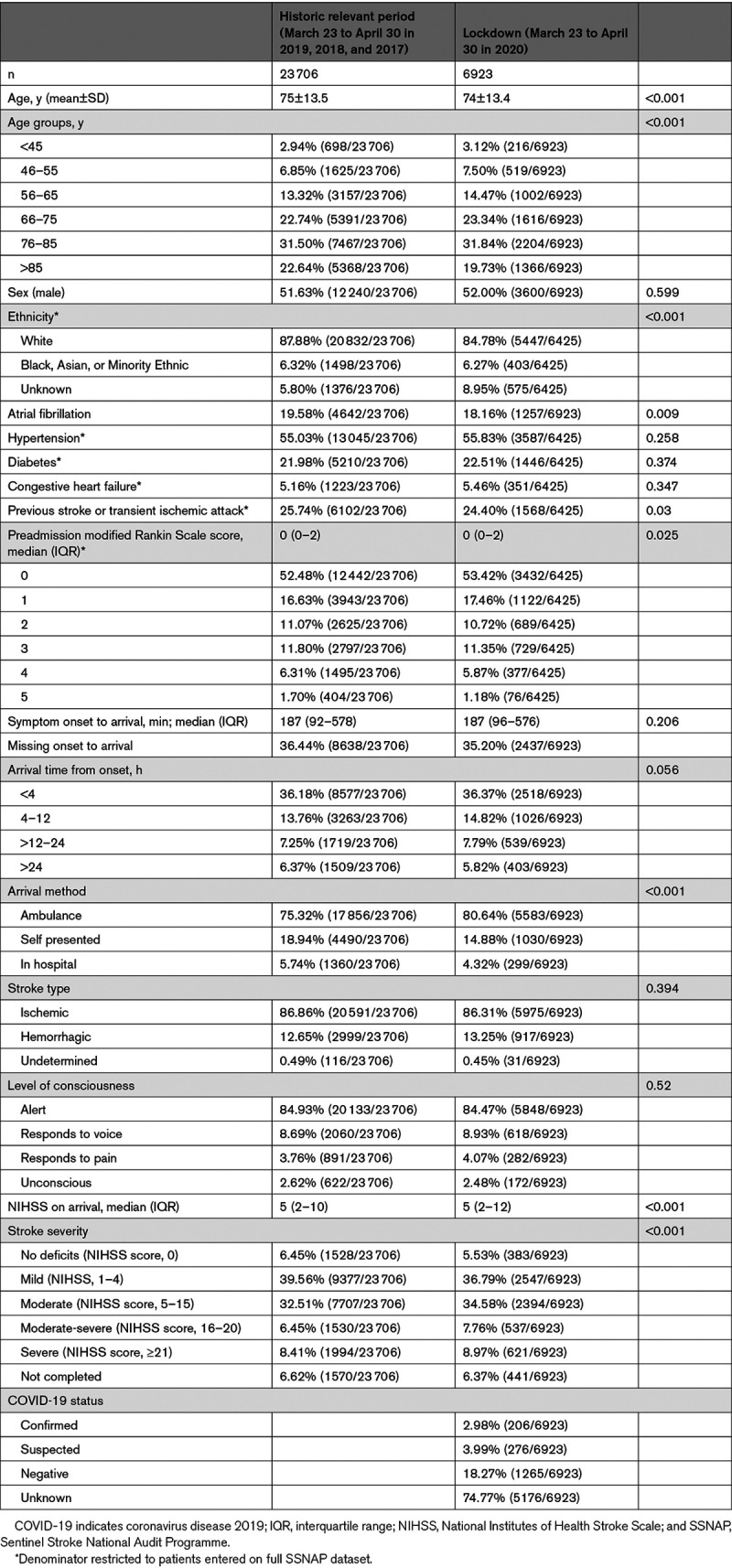
Patient Characteristics

Care quality was maintained or improved for all care quality indicators (Table 2). There was a 9.1% absolute benefit increase (ABI; *P*<0.001) for direct admission to a stroke unit within 4 hours of hospital arrival, a 5.6% ABI (*P*<0.001) for stroke specialist physician assessment within 24 hours, a 5.0% ABI (*P*<0.001) for receiving a brain scan within 1 hour of hospital arrival, a 3.3% ABI (*P*<0.001) for swallow screen within 4 hours of hospital arrival, and a 2.2% ABI in stroke nurse assessment within 24 hours (*P*<0.001). Improvements were also observed in physiotherapy, occupational, and speech and language therapy assessments within 72 hours, although these were offset by a higher proportion of patients being considered ineligible for therapy. Reperfusion treatments were delivered to a similar proportion of patients during lockdown: for intravenous thrombolysis, 13.9% of ischemic strokes were treated (versus 13.3% in 2017–2019), without a significant change in proportions of those treated within 1 hour (59.8% vs 62.7%). Against a background secular trend of increasing mechanical thrombectomy treatment from a low base in the United Kingdom, 2.0% were treated during the 2020 lockdown compared with 1.8% during the same period the previous year.

**Table 2.**
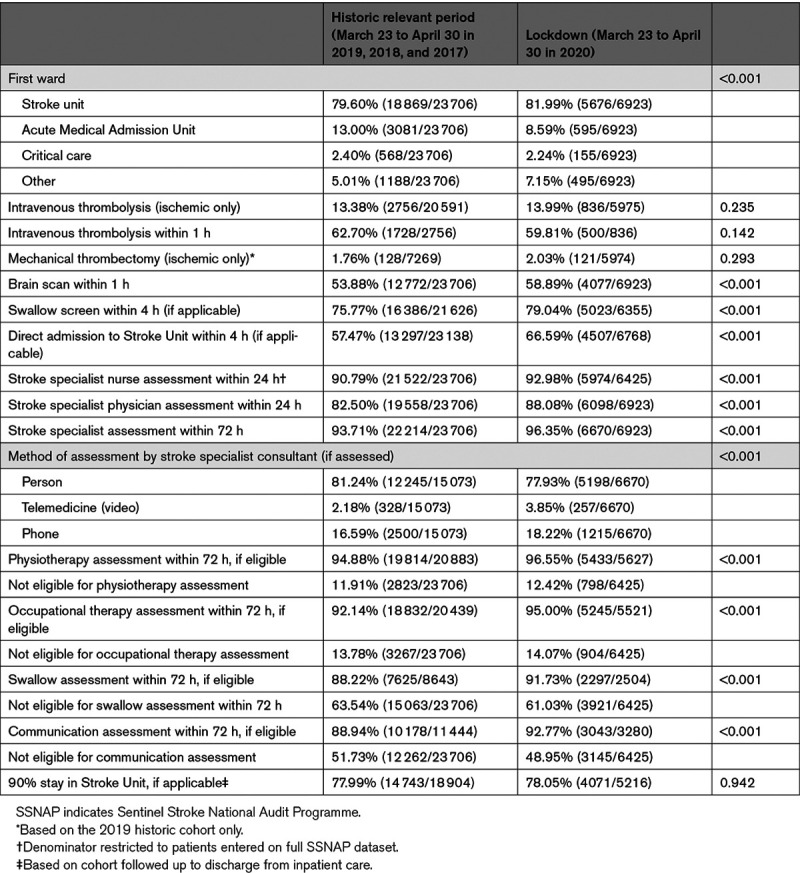
Care Quality Metrics

**Figure 2.**
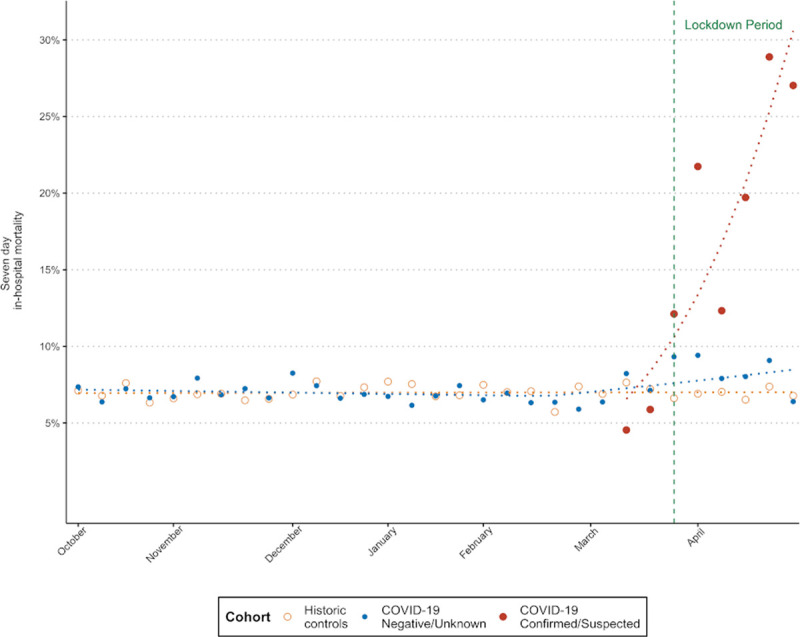
Comparison in trends of 7 d in-hospital mortality between historic controls, patients with coronavirus disease 2019 (COVID-19) status negative/unknown, and patients with confirmed or suspected COVID-19.

Mortality was reported for patients with data locked to hospital discharge, in whom there was a significant increase in 7-day inpatient mortality (9.4% vs 6.9%; *P*<0.001; Table 3; Table II in the Data Supplement), starting from the third week of February (Figure 2). Seven-day mortality was significantly higher in stroke patients with confirmed or suspected COVID-19, at 22.0% and 21.9%, respectively (adjusted rate ratio, 1.41 [1.11–1.80]; *P*<0.006 for confirmed/suspected versus negative/unknown), compared with 7.3% for patients with negative/unknown COVID-19 status (*P*<0.001; Table III in the Data Supplement). During the historic period, the weekly percentage change in 7-day mortality remained stable at 0.03% (95% CI, −0.25 to 0.31). For patients admitted in 2020 with negative or unknown COVID-19 status, the 7-day mortality was comparable to the historic period (−0.33% [95% CI, −1.20 to 0.54]) up to late February when the weekly percentage change increased by 2.4% ([95% CI, 0.33–4.56] Figure 2). For COVID-19–positive patients, the weekly percentage change in 7-day mortality increased by 30% (95% CI, 15.48–46.35).

**Table 3.**
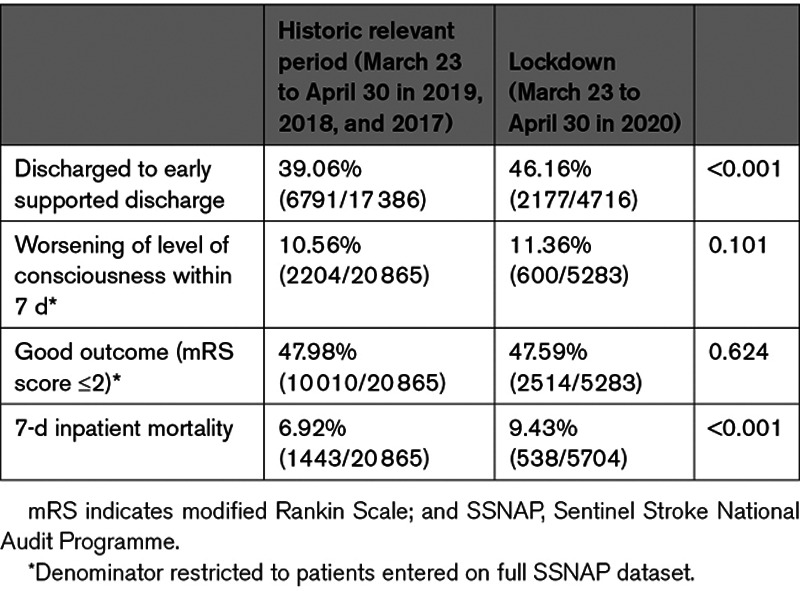
Outcomes

## Discussion

We report how the COVID-19 pandemic has affected stroke care quality and outcomes at a national level in a country with a high infection and mortality rate (311 151 cases and 43 550 deaths in the United Kingdom as of June 28, 2020). While other reports have been published on the impact of COVID-19 on stroke, this study has the advantage of presenting comprehensive information at a national level involving a large cohort of patients, with extensive geographic coverage, including critical aspects of acute care that other reports have not addressed such as access to stroke unit care, speed of screening for dysphagia, and access to rehabilitation therapies.

As reported elsewhere, the immediate impact of the COVID-19 pandemic was to cause a reduction in the number of people presenting to hospital with stroke—an effect that was evident from early February and well before the imposition of population-level lockdown measures. This fall in admissions was predominantly for patients with mild symptoms and particularly in patients over the age of 85 years. Whether this was because there was a reluctance by emergency medical services or primary care to refer patients to hospital due to their increased risk if they did contract coronavirus in an attempt to reduce the burden on the health service or because the patients were not able to alert emergency services themselves or decided against referral is not known. Prehospital routing protocols, although implemented in some regions, were not developed country wide, and there were no explicit changes in stroke triage policies in hospital. The reduction was less marked for hemorrhagic stroke, and it seems probable that the more severe symptoms of intracerebral hemorrhage mean that it is more likely that patients would be referred to hospital even in the context of a pandemic. However, this may be tempered by the lower percentage of patients presenting during lockdown with a previous stroke/transient ischemic attack indicating symptom awareness may not be solely an explanatory factor.

As with previous reports,^9–11^ the quality of care for admitted stroke patients remained high and, in some aspects, improved. The maintenance of reperfusion therapy was welcome, given the complexities of prehospital and hospital pathways in combination with infection control measures. The reasons for the improvements in care quality are likely to be multifactorial. Most planned care in hospitals was suspended at the start of the pandemic in the United Kingdom leading to an increase in the number of available beds even in areas of the country where the number of COVID-19 patients was high, with additional staff drafted in from other clinical areas to work in acute care. In addition to this, there was a national priority given to early discharge to avoid nosocomial infection with a focus on identifying eligible patients as soon as possible. The development of specialist stroke services in every hospital treating stroke patients and the focus in recent years on creating a smaller number of comprehensive stroke centers with better resources, including higher staffing ratios, could have contributed to the ability to maintain high-quality care. In the United Kingdom and some of the other countries that have reported how services have been maintained, one of the common features has been the presence of national stroke plans and strategies for maintaining services during crises.^12–16^

However, despite maintaining high-quality services, the 7-day case fatality rate for stroke increased significantly by 2.5 percentage points. It is not possible to determine whether this higher mortality is explained by the high case fatality rates in the subgroup of patients with confirmed or suspected COVID-19 or as a result of fewer patients with milder stroke being admitted to hospital or a combination of these effects. Another potential effect is a statistical artifact caused by stroke survivors still being in hospital at the time of data extraction and hence not yet having data locked to hospital discharge (a form of collider bias). This observed effect on mortality may require subsequent confirmation when the full dataset to hospital discharge is available.

The strengths of this study are the large sample size drawn from a 4-year period, the comprehensive geographic coverage, and the amount of data on a wide range of quality indicators of patient care, not just in reperfusion treatment rates. The SSNAP dataset is well established with high-quality data resulting both from internal validation within the web-based dataset and through many years of working with clinicians to support data quality in sites. The limitations of the study are that some hospitals did not contribute data during the pandemic and so it is not possible to know whether the findings would be similar in these nonparticipating hospitals. Mortality was ascertained by hospital reporting rather than by linkage to statutory death records, and the relatively short amount of follow-up time currently available means that there is a residual risk of collider bias in the estimates of inpatient mortality due to conditioning on having data locked to discharge. To some extent, this limitation is a consequence of carrying out the study while the COVID-19 pandemic is still ongoing, and data are recent. Future studies using linked data to ascertain deaths and longer follow-up time will enable the causes for the apparent increase in case fatality to be more fully understood, and in the meantime, the study should be considered hypothesis generating.

The COVID-19 pandemic has forced many health care systems to reexamine how care can be delivered more effectively and safely, and these lessons should be carried forward into the post–COVID-19 era. In the meantime, greater use of telemedicine for triaging stroke by emergency medical services before transfer to hospital,^17^ in the emergency department, and subsequently for the delivery of domiciliary rehabilitation and medical follow-up should be developed.^18^ Additional measures are required to ensure that patients with milder stroke who may be avoiding hospital admission are not disadvantaged through inadequate access to early secondary prevention or rehabilitation. In hospital, relatively small increases in bed availability on specialist stroke units can lead to substantially improved access to specialist care for patients with acute stroke and substantial improvements in a range of indicators of care quality.

## Conclusions

The impact of COVID-19 on patients with acute stroke has not been equal, and there may be a hidden cohort of older patients with stroke who did not present to hospital and remained untreated during the pandemic or died from COVID-19. These patients will be at risk of poorer outcomes and higher risk of recurrence. The reasons why the quality of inpatient stroke services was resilient and in some aspects improved during the pandemic should be investigated, and efforts should be made to learn from the unintended positive effects on health services of the pandemic response.

## Acknowledgments

We would like to thank all of the hospital teams that have contributed to Sentinel Stroke National Audit Programme data collection and the patients whose data are included in this study. All authors contributed to study design, provided feedback on the protocol and analysis plan, reviewed and interpreted the results, and contributed to manuscript writing. Dr Bray led the protocol development. Drs Douiri and Muruet analyzed the data. A.G. Rudd, Dr Bray, and Dr Muruet wrote the first draft of the manuscript.

## Sources of Funding

This work was supported by funding from the National Institute for Health Research (NIHR) Applied Research Collaborations South London at King’s College Hospital NHS Foundation Trust and South West Peninsula, with support from the NIHR Biomedical Research Centre based at Guy’s and St Thomas’ NHS Foundation Trust and King’s College London. The Sentinel Stroke National Audit Programme is funded by NHS England, the Welsh Government, and, with some individual projects, other devolved administrations and crown dependencies through the Healthcare Quality Improvement Partnership.

## Disclosures

None.

## Supplemental Materials

Online Tables I–III

Online Figures I–III

RECORD Checklist

## Supplementary Material


