# Supplementary material for: Stroke Care in the United Kingdom During the COVID-19 Pandemic
Source: Stroke. 2021 Apr 26;52(6):2125–33. doi: 10.1161/STROKEAHA.120.032253 (PMC8140645; doi:10.1161/STROKEAHA.120.032253)
Supplement: Supplementary file 1 [file str-52-2125-s001.pdf]

SUPPLEMENTAL MATERIAL

Supplemental Table I

	Trusts participating during COVID-19 (88%, 114/130)	All Trusts (n=130)
Country		
England	92.11% (105/114)	91.54% (119/130)
Wales	4.39% (5/114)	4.62% (6/130)
Northern Ireland	3.51% (4/114)	3.85% (5/130)
Number of patients typically expected to be admitted 23 March - 30 April		
<26	9.65% (11/114)	11.54% (15/130)
26-50	16.67% (19/114)	18.46% (24/130)
51-75	35.09% (40/114)	34.62% (45/130)
76-100	17.54% (20/114)	16.15% (21/130)
101+	21.05% (24/114)	19.23% (25/130)

Supplemental Table II

Seven day mortality by stroke severity

Severity	Historic Relevant Period	Lockdown	p
Not Completed	18.8%	24.1%	0.306
Asymptomatic	0.8%	0.3%	1.000
Mild	0.9%	0.6%	1.000
Moderate	3.8%	5.5%	0.003
Moderate-Severe	16.6%	23.5%	0.003
Severe	36.2%	47.5%	<0.001

Supplemental Table III

	**COVID-19 status**				
	Confirmed	Suspected	None	Unknown	p
Discharged to Early Supported Discharge	26.39% (19/72)	27.59% (32/116)	23.99% (201/838)	51.69% (1,925/3,724)	<0.001
Worsening of Level of Consciousness within 7 Days[1]	23.14% (28/121)	24.39% (40/164)	15.86% (118/744)	9.73% (414/4,254)	<0.001
Good Outcome (modified Rankin Score ≤ 2)[1]	9.09% (11/121)	10.37% (17/164)	46.91% (349/744)	50.24% (2,137/4,254)	<0.001
Seven Day In-patient Mortality	22.01% (35/159)	21.93% (50/228)	13.57% (144/1,061)	7.26% (309/4,256)	<0.001

[1]Denominator restricted to patients entered on full SSNAP dataset

Supplemental Figure I

Weekly percent change in stroke admissions

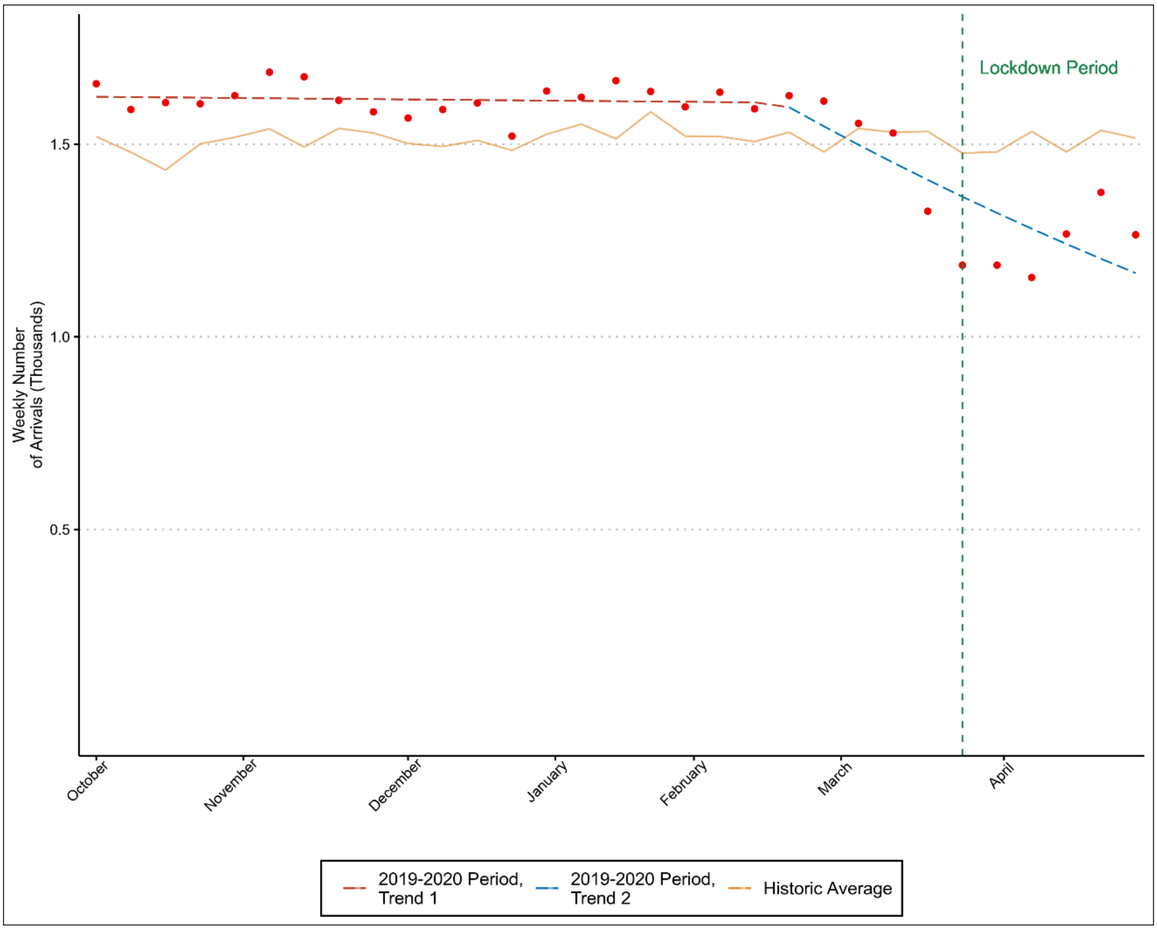

Supplemental Figure II

Weekly number of admissions by age group, compared to the three previous years (dashed lines).

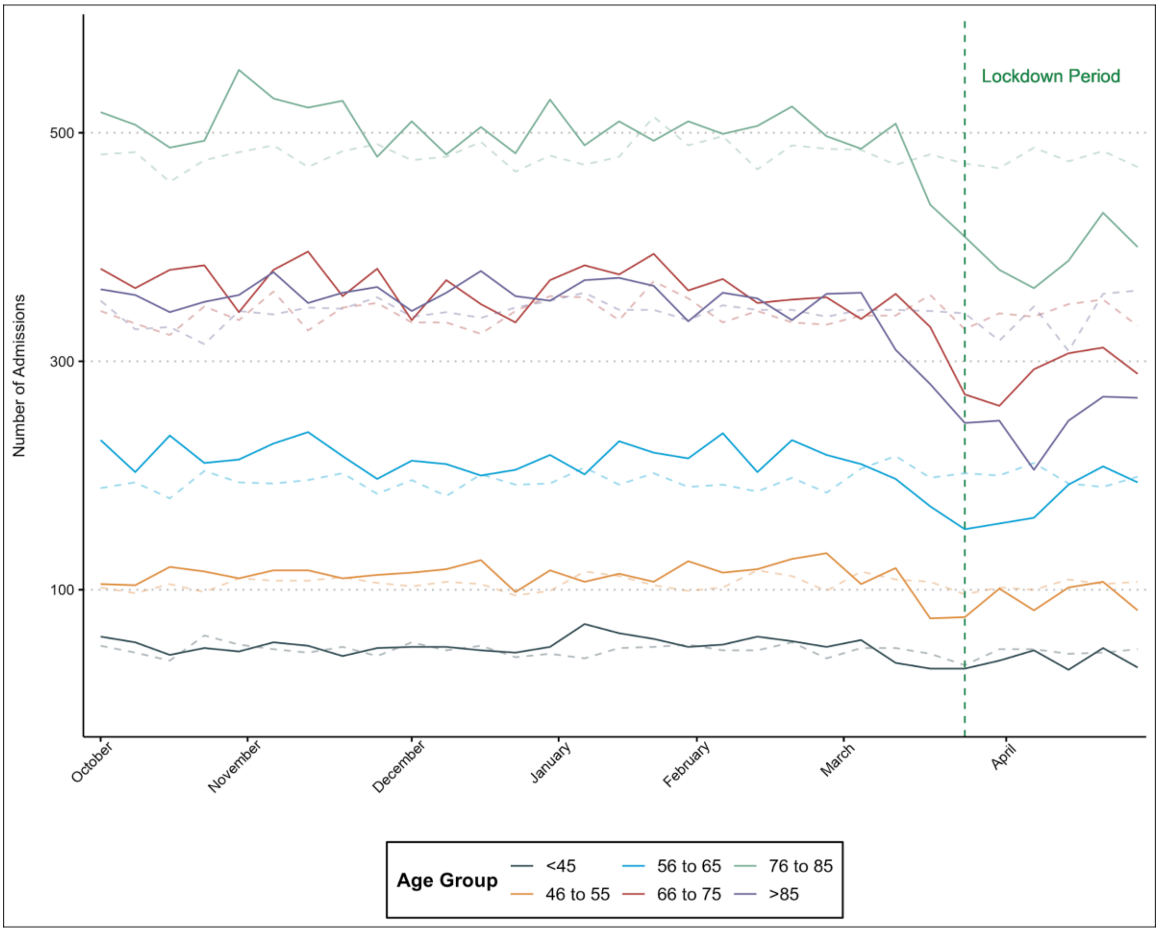

Supplemental Figure III

Weekly number of admissions by stroke severity (NIHSS Score), compared to the three previous years (dashed lines).

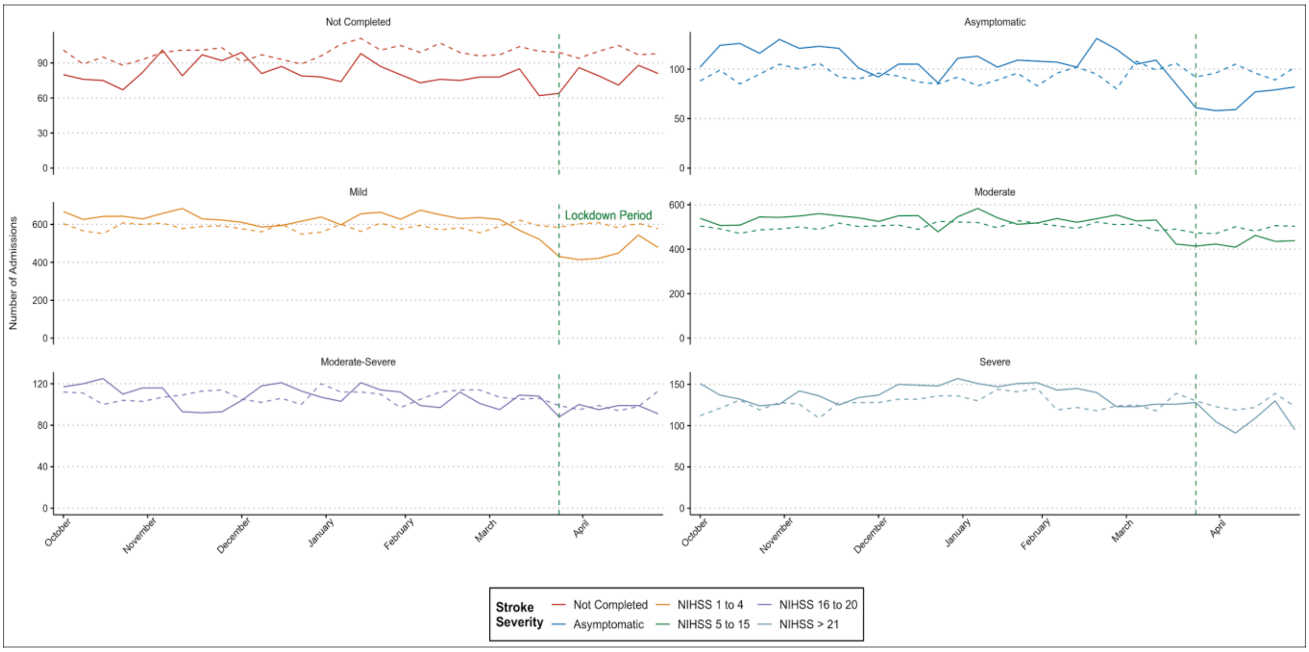

RECORD Checklist

Item No	STROBE items	RECORD items	RECORD-PE items	Response
**Title and abstract**				
1	(a) Indicate the study's design with a commonly used term in the title or the abstract.<br>(b) Provide in the abstract an informative and balanced summary of what was done and what was found.	1.1: The type of data used should be specified in the title or abstract. When possible, the name of the databases used should be included.<br>1.2: If applicable, the geographical region and timeframe within which the study took place should be reported in the title or abstract.<br>1.3: If linkage between databases was conducted for the study, this should be clearly stated in the title or abstract.	—	a) Described in Abstract<br>b) Completed
**Introduction**				
Background rationale				
2	Explain the scientific background and rationale for the investigation being reported.	—	—	Completed om "Introduction" section
Objectives				
3	State specific objectives, including any prespecified hypotheses.	—	—	Described in "Introduction" section
**Methods**				
Study design				
4	Present key elements of study design early in the paper.	—	4.a: Include details of the specific study design (and its features) and report the use of multiple designs if used.<br>4.b: The use of a diagram(s) is recommended to illustrate key aspects of the study design(s), including exposure, washout, lag and observation periods, and covariate definitions as relevant.	Described in "Methods' Study Design"
Setting				
5	Describe the setting, locations, and relevant dates, including periods of recruitment, exposure, follow-up, and data collection.	—	—	Described in "Methods; Data Source"
Participants				

6	(a) Cohort study—give the eligibility criteria, and the sources and methods of selection of participants. Describe methods of follow-up. Case-control study—give the eligibility criteria, and the sources and methods of case ascertainment and control selection. Give the rationale for the choice of cases and controls. Cross sectional study—give the eligibility criteria, and the sources and methods of selection of participants.<br>(b) Cohort study—for matched studies, give matching criteria and number of exposed and unexposed. Case-control study—for matched studies, give matching criteria and the number of controls per case.	6.1: The methods of study population selection (such as codes or algorithms used to identify participants) should be listed in detail. If this is not possible, an explanation should be provided.<br>6.2: Any validation studies of the codes or algorithms used to select the population should be referenced. If validation was conducted for this study and not published elsewhere, detailed methods and results should be provided.<br>6.3: If the study involved linkage of databases, consider use of a flow diagram or other graphical display to demonstrate the data linkage process, including the number of individuals with linked data at each stage.	6.1.a: Describe the study entry criteria and the order in which these criteria were applied to identify the study population. Specify whether only users with a specific indication were included and whether patients were allowed to enter the study population once or if multiple entries were permitted. See explanatory document for guidance related to matched designs.	Described in "Methods; Study Design"
Variables				
7	Clearly define all outcomes, exposures, predictors, potential confounders, and effect modifiers. Give diagnostic criteria, if applicable.	7.1: A complete list of codes and algorithms used to classify exposures, outcomes, confounders, and effect modifiers should be provided. If these cannot be reported, an explanation should be provided.	7.1.a: Describe how the drug exposure definition was developed.<br>7.1.b: Specify the data sources from which drug exposure information for individuals was obtained.<br>7.1.c: Describe the time window(s) during which an individual is considered exposed to the drug(s). The rationale for selecting a particular time window should be provided. The extent of potential left truncation or left censoring	No codelists were required for this study as all the coding is bespoke to the SSNAP registry<br><br>Variables described in "Methods; Statistical Analysis"

			should be specified.<br>7.1.d: Justify how events are attributed to current, prior, ever, or cumulative drug exposure.<br>7.1.e: When examining drug dose and risk attribution, describe how current, historical or time on therapy are considered.<br>7.1.f: Use of any comparator groups should be outlined and justified.<br>7.1.g: Outline the approach used to handle individuals with more than one relevant drug exposure during the study period.	
Data sources/measurement				
8	For each variable of interest, give sources of data and details of methods of assessment (measurement). Describe comparability of assessment methods if there is more than one group.	—	8.a: Describe the healthcare system and mechanisms for generating the drug exposure records. Specify the care setting in which the drug(s) of interest was prescribed.	Described in "Methods; Data Source"
Bias				
9	Describe any efforts to address potential sources of bias.	—	—	Described in Methods
Study size				
10	Explain how the study size was arrived at.	—	—	Not applicable; all available patients meeting the I/E criteria were included
Quantitative variables				
11	Explain how quantitative variables were handled in the analyses. If applicable, describe which groupings were chosen, and why.	—	—	Described in "Methods' Statistical Analysis"
Statistical methods				

12	(a) Describe all statistical methods, including those used to control for confounding.<br>(b) Describe any methods used to examine subgroups and interactions.<br>(c) Explain how missing data were addressed.<br>(d) Cohort study—if applicable, explain how loss to follow-up was addressed. Case-control study—if applicable, explain how matching of cases and controls was addressed. Cross sectional study—if applicable, describe analytical methods taking account of sampling strategy.<br>(e) Describe any sensitivity analyses.	—	12.1.a: Describe the methods used to evaluate whether the assumptions have been met.<br>12.1.b: Describe and justify the use of multiple designs, design features, or analytical approaches.	Described in “Methods’ Statistical Analysis”
Data access and cleaning methods				
12	—	12.1: Authors should describe the extent to which the investigators had access to the database population used to create the study population.<br>12.2: Authors should provide information on the data cleaning methods used in the study.	—	Authors had full access to the anonymised individual patient data
Linkage				
12	—	12.3: State whether the study included person level, institutional level, or other data linkage across two or more databases. The methods of linkage and methods of linkage quality evaluation should be provided.	—	No linkage was carried out
**Results**				
Participants				
13	(a) Report the numbers of individuals at each stage of the study (eg, numbers potentially eligible, examined for eligibility, confirmed eligible, included in the study, completing follow-up, and analysed).<br>(b) Give reasons for non-participation at each stage.<br>(c) Consider use of a flow diagram.	13.1: Describe in detail the selection of the individuals included in the study (that is, study population selection) including filtering based on data quality, data availability, and linkage. The selection of included individuals can be described in the text or by means of the study flow diagram.	—	Described in Results
Descriptive data				
14	(a) Give characteristics of study participants (eg, demographic, clinical, social) and information on exposures and potential confounders.<br>(b) Indicate the number of participants with missing data for each variable of interest.<br>(c) Cohort study—summarise follow-up time (eg, average and total amount).	—	—	Described in Results
Outcome data				
15	Cohort study—report numbers of outcome events or summary measures over time. Case-control study—report numbers in each exposure category, or	—	—	Described in Results

	summary measures of exposure. Cross sectional study—report numbers of outcome events or summary measures.			
Main results				
16	(a) Give unadjusted estimates and, if applicable, confounder adjusted estimates and their precision (eg, 95% confidence intervals). Make clear which confounders were adjusted for and why they were included.<br>(b) Report category boundaries when continuous variables are categorised.<br>(c) If relevant, consider translating estimates of relative risk into absolute risk for a meaningful time period.	—	—	Described in Results
Other analyses				
17	Report other analyses done—eg, analyses of subgroups and interactions, and sensitivity analyses.	—	—	Described in Results
**Discussion**				
Key results				
18	Summarise key results with reference to study objectives.	—	—	Described in "Discussion"
Limitations				
19	Discuss limitations of the study, taking into account sources of potential bias or imprecision. Discuss both direction and magnitude of any potential bias.	19.1: Discuss the implications of using data that were not created or collected to answer the specific research question(s). Include discussion of misclassification bias, unmeasured confounding, missing data, and changing eligibility over time, as they pertain to the study being reported.	19.1.a: Describe the degree to which the chosen database(s) adequately captures the drug exposure(s) of interest.	Described in "Discussion"
Interpretation				
20	Give a cautious overall interpretation of results considering objectives, limitations, multiplicity of analyses, results from similar studies, and other relevant evidence.	—	20.a: Discuss the potential for confounding by indication, contraindication or disease severity or selection bias (healthy adherer/sick stopper) as alternative explanations for the study findings when relevant. **[A: Original text indicated this item was RECORD (ie, not RECORD-PE)?]**	Described in "Discussion; Conclusion"
Generalisability				

21	Discuss the generalisability (external validity) of the study results.	—	—	Described in “Discussion”
**Other information**				
Funding				
22	Give the source of funding and the role of the funders for the present study and, if applicable, for the original study on which the present article is based.	—	—	Described in “Funding” statement
Accessibility of protocol, raw data, and programming code				
22	—	22.1: Authors should provide information on how to access any supplemental information such as the study protocol, raw data, or programming code.	—	

RECORD=reporting of studies conducted using observational routinely collected data; RECORD-PE=RECORD for pharmacoepidemiological research; STROBE=strengthening the reporting of observational studies in epidemiology.

*REFERENCE: Langan SM, Schmidt S, Wing K, Ehrenstein V, Nicholls S, Filion K, Klungel O, Petersen I, Sorensen H, Guttmann A, Harron K, Hemkens L, Moher D, Schneeweiss S, Smeeth L, Sturkenboom M, von Elm E, Wang S, Benchimol EI. The REporting of studies Conducted using Observational Routinely-collected health Data (RECORD) Statement for Pharmacoepidemiology (RECORD-PE). *BMJ* 2018; 363: k3532.
